# Effect of a Single Session of Tai Chi Chuan Practice on Glucose and Lipid Metabolism and Related Hormones

**DOI:** 10.3390/life10080145

**Published:** 2020-08-09

**Authors:** Wan-An Lu, Yung-Sheng Chen, Chun-Hsiung Wang, Cheng-Deng Kuo

**Affiliations:** 1 Institute of Cultural Asset and Reinvention, Fo-Guang University, Yilan 262, Taiwan; wanan.lu@msa.hinet.net; 2 Department of Exercise and Health Sciences, University of Taipei, Taipei 111, Taiwan; yschen@utaipei.edu.tw; 3 Department of Cardiology, Taipei City Hospital Renai Branch, Taipei 106, Taiwan; chjohn.wang@msa.hinet.net; 4 Department of Medical Research, Taipei Veterans General Hospital, Taipei 112, Taiwan; 5 Division of Chest Medicine, Department of Internal Medicine, Changhua Christian Hospital, Changhua 500, Taiwan; 6 Tanyu Research Laboratory, Taipei 112, Taiwan

**Keywords:** serum glucose, insulin resistance, insulin sensitivity, Tai Chi Chuan

## Abstract

Background: To examine the effect of Tai Chi Chuan (TCC) practice on glucose and lipid metabolism and related hormones in TCC practitioners. Methods: Twenty-one TCC practitioners and nineteen healthy controls were included in this study. Classical Yang’s TCC was practiced by the TCC practitioners. The percentage changes in serum total cholesterol (TC), high-density lipoprotein-cholesterol (HDL-C), serum glucose (SG), serum insulin, serum insulin level, homeostatic model assessment of insulin resistance (HOMA-IR), log(HOMA-IR), quantitative insulin sensitivity check index (QUICKI), and serum endothelin-1 (ET-1) before and 30 min after resting or TCC practice were compared between healthy controls and TCC practitioners. Results: Before TCC or resting, the serum insulin level, HOMA-IR, and log(HOMA-IR) of the TCC practitioners were significantly lower than those of healthy subjects, whereas the QUICKI of the TCC practitioners was significantly higher than that of healthy subjects. Thirty min after TCC practice, the %TC, %HDL-C, %QUICKI, and %ET-1 were all significantly decreased, whereas the %SG, %serum insulin, and %HOMA-IR were significantly increased in the TCC group as compared to the control group 30 min after resting. Conclusions: The serum glucose, insulin level and insulin resistance were enhanced, whereas the cholesterol, HDL-C and ET-1 levels were reduced 30 min after TCC practice. The mechanism underlying these effects of TCC 30 min after TCC is not clear yet.

## 1. Introduction

Tai Chi Chuan (TCC) is traditional mind–body calisthenics that has evolved over a history of more than 300 years, yielding many branches or types of TCC. It has been shown that a 12-month TCC training program can significantly improve aerobic capacity and coronary heart disease risk factors in patients with dyslipidemia [1]. An eight-week Tai Chi intervention showed benefits on health status of patients with type 2 diabetes [2]. TCC can be used as an intervention tool to improve glycemic control and serum TG levels in the elderly [3]. Systematic review results indicate that tai chi interventions have a significant and positive effect on blood pressure and lipid levels but not for blood sugar levels [4]. TCC can improve glucose control, balance, neuropathic symptoms, and some dimensions of quality of life in diabetic patients with neuropathy [5]. TCC exercise for three months can reduce the serum levels of endothelin-1 (ET-1) and TG in elderly subjects [6]. TCC can increase the HDL-C and quantitative insulin sensitivity check index (QUICKI) [7], but decrease the serum levels of insulin, homeostasis model assessment-estimated insulin resistance (HOMA-IR), log(HOMA) [8] and ET-1 [9]. A meta-analysis of Tai chi exercise of duration 4 to 24 weeks showed that Tai chi can effectively affect the management of blood glucose and HbA1c in type-2 DM patients, and long-term adherence to TCC has a better role in reducing blood glucose and HbA1c levels in type 2 DM patients [10]. A 3-month Tai Chi exercise improved endothelial dysfunction and arterial stiffness in elderly women with rheumatoid arthritis, suggesting that it can be a useful behavioral strategy for cardiovascular disease prevention in patients with rheumatoid arthritis [11]. However, some studies have reported otherwise. For instance, TCC did not improve existing metabolic syndrome levels, lipid level (dyslipidemia), or fasting glucose level (hyperglycemia) [12], and sufficient evidence to support the benefits of TCC for T2DM patients is lacking [13]. Glucose homeostasis and lipid profile did not show significant changes with TCC compared with the controls over a 24-month period [14]. The effect of TCC on metabolism is still not well understood.

The above-mentioned studies were performed on subjects practicing TCC for 4 to 24 weeks, mostly 12 weeks. No studies regarding the effect of a single bout of TCC exercise on the metabolism can be found in the literature. This study intended to investigate the effects of a single session of TCC exercise on glucose and lipid metabolism and related hormones after a session of TCC practice in TCC practitioners. We hypothesized that a single session of TCC exercise might have an effects on the health of its practitioners through the regulation of glucose and lipid metabolism and related hormones in TCC practitioners.

## 2. Materials and Methods

### 2.1. Subject Selection

Nineteen healthy subjects and twenty-one TCC practitioners were recruited in this study. The TCC practitioners in the study group were recruited from a TCC training center in Taipei, Taiwan, while the healthy subjects without prior TCC experience were recruited from the neighboring community as the control group. All subjects included in this study were over 50 years of age, had ordinary lifestyles, and were able to perform daily activities without restrictions. Subjects who had major cardiac or pulmonary disease or were taking regular medicine for hypertension, diabetes mellitus, and/or liver or renal diseases were not included in this study. The subjects in the control group were the same as those in the study group in every aspect except prior TCC experience.

The sample size was calculated by using G*Power version 3.1.9.4 (G*Power, Düsseldorf, Germany) with serum insulin level as the outcome variable. To identify the group difference, α error probability was set at 0.05 and power (1-β error probability) was set at 0.80. The power estimation indicated that 21 participants in the study group is required.

The Institutional Review Board of the Hospital has approved this study (VGHIRB No. 98-01-59A), and written informed consent was obtained from each participant before the study. This study was registered in ClinicalTrials.gov with identifier NCT03503084.

### 2.2. Study Design

The participants in this study were asked to not consume any alcoholic or caffeinated beverages for at least 24 h before the study, and to not take meal after dinner on the day before the study. They were also requested to not practice TCC or do other kinds of exercise on the day of study. 

After 5 min of rest on the study day, the systolic blood pressure (SBP), diastolic blood pressure (DBP), and pulse pressure (PP) were obtained (OMRON, HEM-770A, OMRON Corporation, Shiokoji Horikawa, Shimogyo-ku, Kyoto, Japan) from every participant in the control and TCC groups. Then a blood sample was withdrawn from the participant for later biochemical assay.

**Figure 1:**
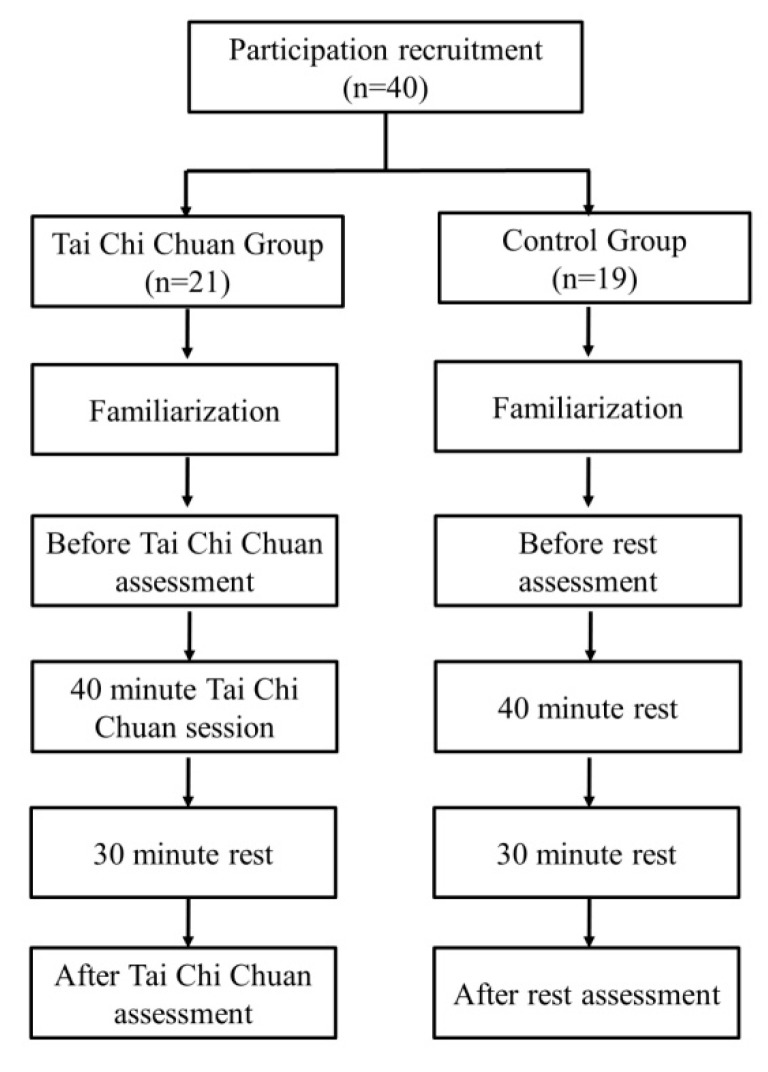
Flow chart of the study.

The TCC practitioners were requested to complete one 40 min session of classical Yang’s TCC. Each Yang’s TCC session included 10 min of warm-up exercises (hamstring and low back stretching, balance training, and gentle calisthenics), 20 min of TCC exercise, and 10 min of cool-down exercise. Each set of Yang’s TCC is composed of 64 successive postures. During TCC exercise, the TCC practitioners maintained the same pace in practicing different postures of TCC according to a pre-recorded tape. Thirty minutes after the completion of TCC exercise in the TCC group or an identical period of rest in the control group, a second measurement of blood pressures and another blood sampling were performed using the same procedure. After the collection of blood using test tube without anticoagulant, the blood was left to clot and the serum was obtained. The serum was then sent to the laboratory and stored in the refrigerator for later biochemical analysis. All procedures were performed in a quiet and bright room with a temperature of 24–25 °C and a humidity of 54–55%. During the rest period, the participants in both groups were requested to sit quietly and comfortably in the seats without conversation with one another. They were allowed to go to the rest room or drink water if necessary. Figure 1 shows the flow chart of the study.

### 2.3. Biochemical and Hormone Analysis

Before the study, the blood samples of the subjects in both groups were collected for the measurements of SG and hormone levels. The length of fast prior to collection of blood samples was at least 12 h because the subjects were requested not to take any meal after 10 pm in the previous night. Thirty minutes after the completion of TCC exercise or an identical period of rest, the blood samples were collected for the measurements of SG and hormone levels. No centrifuge of blood samples was performed because this study used serum for the measurements of biochemical parameters. The blood sample obtained was then sent to the laboratory for the measurement of SG and hormone levels immediately following each collection of blood sample.

The biochemical assays for total cholesterol (TC) (Ektachem Clinical Chemistry Slides, Johnson & Johnson, Johnson & Johnson Plz, New Brunswick, NJ, USA), HDL-C and LDL-C (INTEGRA 700, Roche, Holding AG, Basel, Switzerland), TG, and serum glucose (SG) (Ektachem Clinical Chemistry Slides, Johnson & Johnson, Johnson & Johnson Plz, New Brunswick, NJ, USA) were performed on the serums of the participants before and after the study. The immunoradiometric assay for insulin (DIAsource INS-IRMA Kit, DIAsource ImmunoAssays S.A., Rue du Bosquet 2, 1348 Louvain-La-Neuve, Belgium) and enzyme immunoassay for ET-1 (Kit Lot 998C Abl./Exp. 100908, Biomedica Medizinprodukte GmbH & Co KG, Divischgasse 4 Wien, 1210 Austria) were also performed on the serums from the participants before and after the study.

The insulin resistance and insulin sensitivity of the participants in the fasting state were assessed using HOMA-IR and QUICKI. The HOMA-IR, as the index of insulin resistance, was calculated using the following Equation (1) [15]:

HOMA-IR = fasting insulin (μU/mL) × fasting glucose (mmol/L)/22.5(1)

QUICKI, as the index of insulin sensitivity, was calculated using the following Equation (2) [16]:

QUICKI = 1/(log(fasting insulin in μU/mL) + log(fasting glucose in mg/dL))(2)

The TG/HDL-C was used as another index of insulin resistance in adults [17].

### 2.4. Statistical Analyses

For the comparisons of clinical and biochemical parameters, the percentage changes of the parameters under investigation in each subject prior to and 30 min after a rest or a session of TCC exercise were calculated using the following Equation (3):

%X = ((X_after_ − X_before_)/(X_before_)) × 100% (3)

where X denotes the clinical or biochemical parameter to be compared.

The Mann–Whitney rank sum test (SigmaPlot 14, SPSS Inc., Chicago, IL, USA) was used to compare the parameters between the TCC and control groups. The Wilcoxon signed rank test was used to compare the parameters before and 30 min after TCC exercise or rest in the TCC and control groups.

The baseline characteristics of healthy controls and TCC practitioners are expressed as mean ± SD. The parameters under investigation are presented as median and interquartile range (25% to 75%). A *p* < 0.05 was regarded as statistically significant.

## 3. Results

### Between-Group and Intra-Group Comparisons

Table 1 lists the baseline characteristics of healthy controls and TCC practitioners. There were no significant differences in all baseline characteristics between the two groups of subjects except the length of TCC training.

Between-group comparison showed that the baseline TG, insulin, HOMA-IR, and log(HOMA-IR) of the TCC group were significantly lower than those of the control group, whereas the QUICKI of the TCC group was significantly higher than that of the control group (Table 2). Thirty minutes after resting in the control group or a session of TCC exercise in the TCC group, the SG of the TCC practitioners was significantly higher than that of the control group, whereas the ET-1 of the TCC practitioners was significantly lower than that of the control group.

Intra-group comparison shows that 30 min after resting the DBP, MABP, TC, LDL-C, HDL-C, and QUICKI were significantly increased in the control group (Table 2). In the TCC group, the insulin, HOMA-IR, log(HOMA) and QUICKI were significantly increased, whereas the ET-1 was significantly decreased 30 min after TCC exercise (Table 2). Since many clinical parameters were changed during rest in the control group, the comparisons between the TCC group and the control group were made by comparing the percentage changes in the clinical parameters under investigation so that the change in these clinical parameters shortly after TCC can be realized.

Table 3 shows that 30 min after TCC exercise, the %SG, %insulin and %HOMA-IR were significantly increased, the %TC, %HDL-C, %QUICKI, and %ET-1 were significantly decreased, but the %log(HOMA-IR) was not significantly changed, in the TCC group, as compared to the control group 30 min after resting. This bewildering result suggested that TCC exercise might consume more glucose and insulin and induce more increase in insulin resistance, and consume less TC and HDL-C and induce less increase in insulin sensitivity in the TCC practitioners 30 min after TCC. Table 3 also showed that ET-1 was significantly decreased 30 min after TCC practice as compared to the control group.

Table 4 shows that there were no significant differences in the percentage changes in the parameters of blood pressure, lipid profile, insulin resistance, and ET-1 after a single session of TCC between the male and female TCC practitioners.

## 4. Discussion

Before TCC exercise, the insulin, HOMA-IR, and log(HOMA-IR) of the TCC practitioners were significantly lower than those of the healthy controls, whereas the QUICKI of the TCC practitioners was significantly higher than that of the healthy controls.

Thirty minutes after a session of TCC exercise, the %SG, %Insulin and %HOMA-IR were significantly greater and the %TC, %HDL-C, %QUICKI, and %ET-1 were significantly lower in the TCC group, as compared with the healthy controls after resting. This finding suggested that shortly after TCC exercise there was an increase in serum glucose, insulin level and insulin resistance, plus a decrease in serum TC and HDL-C levels, serum ET-1 level, and insulin sensitivity in TCC practitioners.

### 4.1. Effect of TCC on Glucose and Insulin Levels

Yap et al. [18] demonstrated that 30 min brisk walking was sufficient to improve the insulin sensitivity of healthy young Asians. We found in this study that there was an increase in insulin resistance and a decrease in insulin sensitivity 30 min after TCC exercise. It is not clear why the serum glucose, insulin level, and insulin resistance were increased shortly after TCC exercise, instead of being decreased because of increased consumption due to tissue demand. This bewildering result might be caused by the imbalance between glucose and insulin production and their consumption due to tissue demand. After the initiation of TCC exercise, the glucose is produced and the insulin is secreted so that the muscle can use the glucose to perform TCC exercise. Since TCC is a slow-paced and gentle exercise, not much glucose and insulin are needed for its practice. Therefore, the glucose and insulin consumed during TCC exercise might be less than their production and secretion, resulting in the accumulation of glucose and insulin in the blood stream. This may explain why there was an increase in serum glucose, insulin level and insulin resistance, and a decrease in insulin sensitivity in TCC practitioners 30 min after TCC exercise. Further studies are needed to confirm this finding and to investigate the underlying mechanism.

### 4.2. Effect of TCC on Lipid Metabolism

The lipid panel assayed in this study includes TC, LDL-C, HDL-C, and TG. Some lipids have strong associations with the extent of coronary artery disease [19]. Magkos [20] reported that exercise-induced hypotriglyceridemia is acute and short-lived because it becomes evident 12–18 h after a single session of exercise and could last two to three days. Jafari et al. [21] showed that exercise could significantly increase the plasma level of prebeta-1 HDL and decrease the plasma level of HDL-TG. In this study we found that 30 min after TCC exercise both TC and HDL-C were decreased in the TCC group while the TG, HDL-C/TC and TG/HDL-C were not significantly different, as compared with the healthy controls after resting. Our findings were not the same as previous reports because the comparison was not on the same footing. Further studies are needed to illustrate the effects of TCC on lipid metabolism. The mechanism of decrease in cholesterol and HDL-C shortly after TCC exercise is not clear yet.

### 4.3. Effect of TCC on ET-1 Level

Endothelial function is the result of balanced interaction between vascular cell protectors and risk factors. Under physiological conditions, the vascular endothelium has an anti-thrombogenic function. The ET-1 is involved in the pathogenesis of hypertension and atherosclerosis [22]. Circulating ET-1 might act as a paracrine factor in the metabolism of serum glucose via the modulation of serum insulin level [23]. An elevated ET-1 plasma level has been observed in patients with essential hypertension, atherosclerosis [22], diabetes [23], myocardial infarction [24], and heart failure. Maeda et al. reported that regular aerobic-endurance exercise can reduce plasma ET-1 concentrations in aged people [25]. Lewczuk et al. [26] demonstrated that cycling can decrease plasma ET-1 concentration in healthy men. In accordance with these studies, we found that the serum ET-1 level of TCC practitioners was decreased 30 min after TCC exercise. Since serum ET-1 level is associated with disease severity [27], the decrease in serum ET-1 level due to TCC exercise might be beneficial to the cardiovascular and metabolic functioning of the TCC practitioners.

### 4.4. Changes in Blood Pressure

We accidentally found that both DBP and MABP were increased in the sedentary control subjects after resting, suggesting that sedentary rest for as short as 70 min (40 min + 30 min) may not be good for cardiovascular health. Sedentary activity and lifestyle have been found to be a risk factor for cardiovascular disease and mortality [28], probably because they modify the key hemodynamic, inflammatory, and metabolic processes, resulting in impaired arterial health [29].

### 4.5. Limitations

The first limitation of this study is that no cross-over design was adopted to disclose more specifically what has been changed in glucose and lipid metabolism after a session of TCC. The second limitation of this study is that no blood sample was taken immediately after TCC so that the change in glucose and lipid metabolism right after TCC exercise cannot be revealed. The third limitation of this study is that it was a small scale study only. Further studies are needed to confirm the findings in this study. The fourth limitation is that this study did not include other exercises for comparison, such as walking, jogging, running, and football. The findings observed in this study needs comparison with those after other kinds of exercise so that the meaning of the findings in this study can be appreciated. The fifth limitation the hematocrit was not measured to correct serum insulin, glucose, etc. for the changes in hematocrit. This is a confounding factor that could skew the results if not corrected for. Finally, the sixth limitation of this study is that no further studies were performed to investigate the underlying mechanism of the findings observed in this study. This also must be done in the future studies.

## 5. Conclusions

The current study suggests that the serum glucose, serum insulin level and insulin resistance were enhanced, whereas the cholesterol, HDL-C and ET-1 levels were reduced 30 min after TCC practice. The mechanism of the effect of TCC on metabolism 30 min after TCC is not clear yet. Further studies are warranted to confirm this finding and explore the underlying mechanism.

## Figures and Tables

**Table 1:** Baseline characteristics of normal controls and Tai Chi Chuan (TCC) practitioners.

Characteristics	Normal Controls (*n* = 19)	TCC Practitioners (*n* = 21)
Age (years)	51.8 ± 7.3	54.8 ± 9.3
Sex (M/F)	9/10	12/9
Body weight (kg)	65.0 ± 8.2	63.2 ± 10.4
Body height (cm)	164.0 ± 5.3	164.8 ± 8.3
BMI (kg/m^2^)	24.1 ± 2.7	23.2 ± 2.5
Waist (cm)	84.4 ± 9.7	82.0 ± 9.7
Length of TCC training (months)	0	3.0 ± 0.0 *

Data are presented as mean ± SD. BMI, body mass index. * *p* < 0.05 vs. normal controls.

**Table 2:** Blood pressure, lipid profile, serum glucose (SG), and endothelin-1 (ET-1) of normal controls and TCC practitioner before and 30 min after TCC exercise.

Variables	Controls (*n* = 19)		TCC Practitioners (*n* = 21)	
	Before Rest	After Rest	Before TCC	After TCC
**Blood pressure**				
SBP (mmHg)	114 (109–129)	125 (116–134)	123 (115–140)	126 (112–133)
DBP (mmHg)	73 (62–79)	78 (70–87) *	77 (68–88)	77 (71–83)
MABP (mmHg)	89 (78–94)	95 (86–104) *	92 (83–102)	93 (85–99)
PP (mmHg)	44 (38–51)	43 (39–58)	46 (38–56)	47 (39–51)
**Lipid profile**				
TG (mg/dL)	147 (118–206)	168 (90–259)	102 (78–167) ^a^	108 (83–162)
TC (mg/dL)	178 (143–198)	182 (173–207) *	186 (165–203)	188 (158–210)
LDL-C (mg/dL)	108 (75−121)	121 (99−139) *	118 (100−130)	114 (92–139)
HDL-C (mg/dL)	47 (43–54)	51 (48–63) *	52 (46–62)	57 (19–67)
HDL-C/TC	0.27 (0.24–0.34)	0.27 (0.25–0.35)	0.27 (0.25–0.34)	0.29 (0.26–0.36)
TG/HDL-C	3.14 (2.52–4.54)	2.78 (1.75–5.32)	1.93 (1.19–3.27)	1.93 (1.35–3.27)
**Insulin resistance and ET-1**				
SG (mmol/L)	4.4 (4.2–5.0)	4.2 (4.0–4.5)	4.3 (4.1–5.2)	4.7 (4.4–5.2) ^b^
Insulin (µU/mL)	2.6 (1.1–5.0)	1.4 (0.6–3.7)	1.3 (0.4–2.2) ^a^	2.5 (1.4–3.8) ^†^
HOMA-IR	0.53 (0.21–0.99)	0.24 (0.1–0.69)	0.26 (0.08–0.42) ^a^	0.54 (0.29–1.05) ^†^
log(HOMA-IR)	−0.27 (−0.67 to 0.0)	−0.62 (−1.0 to −0.16)	–0.59 (−1.09 to −0.38) ^a^	−0.27 (−0.54 to 0.02)^†^
QUICKI	0.43 (0.38–0.52)	1.37 (0.84–2.85) *	0.50 (0.45–0.66) ^a^	0.93 (0.73–1.23) ^†^
ET-1 (fmol/mL)	0.58 (0.38–1.23)	0.87 (0.42–1.44)	0.79 (0.42–1.12)	0.38 (0.31–0.64) ^†,^^b^

Data are presented as medians (25–75%). SBP, systolic blood pressure; DBP, diastolic blood pressure; MABP, mean arterial blood pressure; PP, pulse pressure; TG, triglycerides; TC, total cholesterol; LDL-C, low-density lipoprotein-cholesterol; HDL-C, high-density lipoprotein-cholesterol; HDL-C/TC, ratio of HDL-C to TC; TG/HDL-C TG to HDL-C ratio; SG, serum glucose; ET-1, endothelin-1; HOMA-IR, homeostasis model assessment-estimated insulin resistance; QUICKI, quantitative insulin sensitivity check index. Pre-post comparison: * *p* < 0.05 vs. control before rest or TCC; ^†^ *p* < 0.05 vs. TCC practitioner before TCC. Inter-group comparison: ^a^ *p* < 0.05 vs. control before rest; ^b^ *p* < 0.05 vs. control after rest.

**Table 3:** Comparison of percentage changes in study parameters between normal controls and TCC practitioner 30 min after rest or exercise.

Variables	Controls (*n* = 19)	TCC Practitioners (*n* = 21)	*p* Value
**Blood pressure**			
%SBP	4.6 (−3.1 to 15.5)	−2.4 (−8.8 to 4.3)	0.123
%DBP	6.3 (−2.8 to 23.3)	1.2 (−7.8 to 8.2)	0.267
%MABP	5.8 (−5.7 to 19.8)	0.4(−6.6 to 7.0)	0.159
%PP	1.8 (−10.6 to 21.4)	−5.8 (−17.3 to 9.5)	0.244
**Lipid profile**			
%TG	3.7 (−22.2 to 18.7)	−7.8 (−28.5 to 29.0)	0.448
%TC	8.6 (−1.0 to 22.9)	4.0 (−8.4 to 13.8)	0.046 *
%LDL-C	17.4 (−2.6 to 43.3)	7.4 (−14.2 to 20.8)	0.051
%HDL-C	10.5 (2.8 to 24.2)	4.3 (−0.2 to 11.1)	0.029 *
%HDL-C/TC	1.0 (−6.2 to 8.4)	5.6 (−5.7 to 15.2)	0.364
%TG/HDL-C	−3.4 (−39.9 to 14.8)	−9.5 (−33.5 to 32.6)	0.807
**Insulin resistance and ET-1**			
%SG	−6.6 (−14.8 to 1.6)	4.6 (−8.4 to 18.5)	0.028 *
%Insulin	−2.2 (−76.0 to 81.2)	170.0 (31.4 to 346.3)	0.009 *
%HOMA-IR	−10.3 (−77.2 to 77.1)	190.3 (15.0 to 432.3)	0.009 *
%log(HOMA-IR)	−16.6 (−76.4 to 282.7)	−69.4 (−107.2 to 13.6)	0.136
%QUICKI	190.8 (82.2 to 424.6)	70.7 (40.3 to 140.7)	0.017 *
%ET-1	28.3 (−62.9 to 200.0)	−38.7 (−66.4 to −8.1)	0.023 *

Data are presented as medians (25–75%). %X, %X = ((X_after_−X_before_)/(X_before_)) × 100, where X denotes the clinical or biochemical parameter to be compared. * *p* < 0.05, Mann–Whitney Rank sum test.

**Table 4:** Comparison of percentage changes in study parameters between male and female TCC practitioner 30 min after exercise.

Variable	TCC Male (*n* = 12)	TCC Female (*n* = 9)	*p* Value
**Blood pressure**			
%SBP	−3.1 (−8.8 to 5.8)	−5.1 (−8.9 to 4.0)	0.123
%DBP	6.3 (−2.8 to 23.3)	1.2 (−7.8 to 8.2)	0.522
%MABP	−2.0 (−8.5 to 6.7)	0.4(−4.3 to 4.6)	0.619
%PP	−8.0 (−17.5 to 7.1)	−5.8 (−21.3 to 3.9)	0.522
**Lipid profile**			
%TG	−9.6 (−33.0 to 30.5)	−4.3 (−33.9 to 36.3)	0.887
%TC	5.5 (−10.8 to 13.9)	−1.9 (−12.6 to 13.1)	0.570
%LDL-C	14.8 (−3.6 to 28.4)	−11.7 (−19.9 to 11.3)	0.055
%HDL-C	4.7 (0.6 to 9.7)	2.0 (−3.9 to 10.3)	0.619
%HDL-C/TC	6.4 (−7.1 to 14.9)	9.8 (−5.5 to 17.1)	0.722
%TG/HDL-C	−11.9 (−34.9 to 30.2)	−5.2 (−36.7 to 37.0)	0.831
**Insulin resistance and ET-1**			
%SG	5.6 (−8.7 to 10.6)	2.5 (−13.4 to 11.5)	0.569
%Insulin	278.2 (53.4 to 513.4)	104.9 (−24.6 to 267.6)	0.177
%HOMA-IR	271.8 (89.9 to 617.8)	127.7 (−30.1 to 432.3)	0.227
%log(MOMA-IR)	−60.9 (−112.2 to −34.7)	−31.7 (−88.9 to 114.7)	0.570
%QUICKI	56.4 (20.2 to 105.3)	70.7 (40.3 to 186.5)	0.155
%ET-1	−63.1 (−69.5 to v8.0)	−38.7 (−69.4 to −20.0)	1.000

Data are presented as medians (25–75%). %X, %X = ((X_after_ − X_before_)/(X_before_)) × 100, where X denotes the clinical or biochemical parameter to be compared.
